# Supplementary material for: The evolution of a super-swarm of foot-and-mouth disease virus in cattle
Source: PLoS One. 2019 Apr 25;14(4):e0210847. doi: 10.1371/journal.pone.0210847 (PMC6483180; doi:10.1371/journal.pone.0210847)
Supplement: S1 Fig — (PDF) [file pone.0210847.s001.pdf]

Arzt et al. FMDV evolution in cattle

Supplementary Figure 1. Alignment of 52 consensus virus sequences† with inoculum consensus as reference

Genome region		Lpro														VP4	VP2																
Nucleotide position		10	32	39	55	95	100	165	168	234	276	369	381	436	543	816	873	972	988	1029	1032	1068	1102	1107	1120	1131	1160	1250	1263	1275	1380	1425	1441
Inoculum consensus		A	T	T	G	C	C	C	C	C	A	G	G	C	T	C	G	A	G	G	A	A	G	C	C	T	T	A	G	A	T	C	G
Non-vaccinated cattle	14-20 1 dpi Np																																
	14-25 3 dpi Np																																
	14-25 3 dpi Ves																																
	14-25 3 dpi Se																																
	14-34 4 dpi Ns																																
	14-34 4 dpi Sa																																
	14-34 5 dpi Sa																																
	14-34 6 dpi Sa																																
	14-34 7 dpi Sa												A																				
	14-34 8 dpi Sa												A																				
	14-34 9 dpi Sa												A																				
	14-34 17 dpi OPF								T																								
	14-34 35 dpi OPF								T																								
	14-34 35 dpi Np		C						T		G																		A				
	14-44 4 dpi Ves																													G			
	14-44 4 dpi Np																																
	14-44 4 dpi Se																																
	14-47 2 dpi Sa																																
	14-49 2 dpi Sa																																
	14-49 6 dpi Se									T								G															
	14-49 10 dpi Np																																A
	14-110 6 dpi Se																																
	14-110 6 dpi Ns																																
	14-110 8 dpi Sa																																
	14-110 14 dpi OPF																		A														
	14-110 17 dpi OPF			C															A	A													
	14-110 21 dpi OPF																		A														
	14-110 24 dpi OPF																		A														
	14-110 28 dpi OPF																		A														
	14-110 31 dpi OPF																A		A							C							
	14-110 35 dpi Np																		A														
	14-110 35 dpi OPF																		A														
Vaccinated cattle	14-53 3 dpi Np									T								G															
	14-54 14 dpi OPF																																
	14-54 35 dpi Np						T																										
	14-54 35 dpi OPF						T																										
	14-112 1 dpi Np																																
	15-12 3 dpi Sa																																
	15-12 5 dpi Sa														C																		
	15-12 14 dpi OPF																				G		A								C		
	15-12 21 dpi OPF		G											G									A								C		
	15-12 28 dpi OPF							T																	A							T	
	15-12 35 dpi OPF				A			T								T									A							T	
	15-13 7 dpi OPF																					G											
	15-13 14 dpi OPF										G																						
	15-13 21 dpi OPF											C										G									C		
	15-13 28 dpi OPF																					G									C		
	15-13 35 dpi OPF																							T				G					
	15-14 5 dpi Ns																										C						
	15-14 17 dpi OPF					T																										T	
	15-14 28 dpi OPF					T																											
Amino acid change*		4	11		19	32	34					123		146					44				82		88		101	131					195
Genome region		Lpro														VP4	VP2																

Genome region		VP3																								
Nucleotide position		1539	1542	1599	1617	1656	1665	1720	1721	1736	1755	1767	1779	1807	1833	1860	1903	1904	1928	1959	2036	2048	2080	2094	2136	2157
Inoculum consensus		C	T	G	T	C	T	G	A	C	A	C	A	A	G	T	G	A	G	C	C	T	A	G	A	G
Non-vaccinated cattle	14-20 1 dpi Np				C												A									
	14-25 3 dpi Np										T	T														
	14-25 3 dpi Ves										T	T														
	14-25 3 dpi Se										T	T														
	14-34 4 dpi Ns										T	T														
	14-34 4 dpi Sa										T	T														
	14-34 5 dpi Sa										T	T														
	14-34 6 dpi Sa								C		T	T														
	14-34 7 dpi Sa										T	T														
	14-34 8 dpi Sa										T	T														
	14-34 9 dpi Sa										T	T														
	14-34 17 dpi OPF			A																						
	14-34 35 dpi OPF			A		T																				
	14-34 35 dpi Np			A															A				G			
	14-44 4 dpi Ves				C												A									
	14-44 4 dpi Np				C												A									
	14-44 4 dpi Se																									
	14-47 2 dpi Sa										T	T														
	14-49 2 dpi Sa										T	T														
	14-49 6 dpi Se										T	T														
	14-49 10 dpi Np		T					C				T	T											A		
	14-110 6 dpi Se										T	T														
	14-110 6 dpi Ns										T	T														
	14-110 8 dpi Sa										T	T														
	14-110 14 dpi OPF				C												A									
	14-110 17 dpi OPF				C												A								G	
	14-110 21 dpi OPF							A			T	T		G												
	14-110 24 dpi OPF							A			T	T		G												
	14-110 28 dpi OPF							A			T	T		G												
	14-110 31 dpi OPF							A			T	T		G												
	14-110 35 dpi Np							A			T	T		G												
	14-110 35 dpi OPF							A			T	T		G												
Vaccinated cattle	14-53 3 dpi Np										T	T														
	14-54 14 dpi OPF										T	T														
	14-54 35 dpi Np										T									T		C				
	14-54 35 dpi OPF										T	T														
	14-112 1 dpi Np										T	T														
	15-12 3 dpi Sa										T	T				C										
	15-12 5 dpi Sa										T	T									A					
	15-12 14 dpi OPF										T	T														
	15-12 21 dpi OPF										T	T														
	15-12 28 dpi OPF			C								T	T													A
	15-12 35 dpi OPF										T	T			A											A
	15-13 7 dpi OPF				C								T				A									
	15-13 14 dpi OPF										T	T						G								
	15-13 21 dpi OPF				C								T				A									
	15-13 28 dpi OPF				C								T				A									
	15-13 35 dpi OPF										T	T						G								
	15-14 5 dpi Ns										T	T									A					
	15-14 17 dpi OPF					T				T	T	T														
	15-14 28 dpi OPF					T					T	T														
Amino acid change*								70		75				99			131		139			175	179		190	
Genome region		VP3																								

Genome region		VP1																						2A	2B									
Nucleotide position		2178	2241	2258	2259	2269	2356	2379	2401	2415	2461	2577	2589	2595	2605	2607	2615	2639	2654	2676	2762	2765	2771	2816	2901	2910	2916	2958	2996	3168	3183	3198	3259	3354
Inoculum consensus		C	A	A	C	A	C	A	C	A	T	T	G	T	A	T	C	T	C	C	C	C	A	A	G	C	C	C	C	C	C	C	T	C
Non-vaccinated cattle	14-20 1 dpi Np																																	
	14-25 3 dpi Np																T																	
	14-25 3 dpi Ves																T											T						
	14-25 3 dpi Se																T																	
	14-34 4 dpi Ns		G														T																	
	14-34 4 dpi Sa		G														T																	
	14-34 5 dpi Sa		G														T																	
	14-34 6 dpi Sa		G														T																	
	14-34 7 dpi Sa							C							C																			
	14-34 8 dpi Sa							C			A				C						T													
	14-34 9 dpi Sa							C							C																			
	14-34 17 dpi OPF														C																			
	14-34 35 dpi OPF														C																			
	14-34 35 dpi Np														C				T															
	14-44 4 dpi Ves																																	
	14-44 4 dpi Np																																	
	14-44 4 dpi Se																																	
	14-47 2 dpi Sa		G														T																	
	14-49 2 dpi Sa																T																	
	14-49 6 dpi Se													C			T																	
	14-49 10 dpi Np				A			C							C							T	G											
	14-110 6 dpi Se			G					T								T												T					
	14-110 6 dpi Ns			G					T								T												T					
	14-110 8 dpi Sa			G					T								T												T					
	14-110 14 dpi OPF											C				A	T																	
	14-110 17 dpi OPF						T					C				A	T																	
	14-110 21 dpi OPF		G													A	T														T			
	14-110 24 dpi OPF		G													A	T														T			
	14-110 28 dpi OPF		G													A	T														T			
	14-110 31 dpi OPF		G													A	T										T				T		C	
	14-110 35 dpi Np		G													A	T														T			
	14-110 35 dpi OPF		G													A	T														T			
Vaccinated cattle	14-53 3 dpi Np	T															T														T			
	14-54 14 dpi OPF															A	T																	
	14-54 35 dpi Np		G													A	T								A									
	14-54 35 dpi OPF		G													A	T																	
	14-112 1 dpi Np																T																	
	15-12 3 dpi Sa																T																	
	15-12 5 dpi Sa																T																	T
	15-12 14 dpi OPF																T																	
	15-12 21 dpi OPF																T									T								
	15-12 28 dpi OPF									G							T	C												T				
	15-12 35 dpi OPF									G							T	C																
	15-13 7 dpi OPF																T							G										
	15-13 14 dpi OPF		G														T						G											
	15-13 21 dpi OPF																T			T				G										
	15-13 28 dpi OPF																T							G										
	15-13 35 dpi OPF		G										A				T															T		
	15-14 5 dpi Ns																T																	T
	15-14 17 dpi OPF					G										A	T																	
	15-14 28 dpi OPF															A	T																	
Amino acid change*				28		32					96				144		147	155	160		196	197	199	3					45					
Genome region		VP1																						2A	2B									

Genome region		2C																								
Nucleotide position		3363	3414	3452	3480	3495	3534	3541	3574	3591	3633	3645	3663	3732	3747	3750	3804	3995	3999	4067	4068	4131	4134	4137	4171	4225
Inoculum consensus		C	C	C	A	C	C	G	C	C	C	C	T	T	C	G	C	A	G	T	C	C	C	C	G	A
Non-vaccinated cattle	14-20 1 dpi Np																								A	
	14-25 3 dpi Np																									
	14-25 3 dpi Ves																									
	14-25 3 dpi Se																									
	14-34 4 dpi Ns																							T		
	14-34 4 dpi Sa																							T		
	14-34 5 dpi Sa																							T		
	14-34 6 dpi Sa																							T		
	14-34 7 dpi Sa				T					A					G						C			T		A	
	14-34 8 dpi Sa				T	G			A	A					G					A	C			T		A	
	14-34 9 dpi Sa				T					A		T			G						C			T		A	
	14-34 17 dpi OPF																			A						A	
	14-34 35 dpi OPF																			A						A	
	14-34 35 dpi Np																	T		A						A	
	14-44 4 dpi Ves																								A	
	14-44 4 dpi Np																								A	
	14-44 4 dpi Se																								A	
	14-47 2 dpi Sa																							T		
	14-49 2 dpi Sa																									
	14-49 6 dpi Se																									
	14-49 10 dpi Np				T					A					G						C			T		A	
	14-110 6 dpi Se																									
	14-110 6 dpi Ns																									
	14-110 8 dpi Sa																									
	14-110 14 dpi OPF																								A	
	14-110 17 dpi OPF														T										A	
	14-110 21 dpi OPF											T	C											T		
	14-110 24 dpi OPF											T	C											T		
	14-110 28 dpi OPF											T	C											T		
	14-110 31 dpi OPF											T	C											T		
	14-110 35 dpi Np											T	C											T		
	14-110 35 dpi OPF											T	C											T		
Vaccinated cattle	14-53 3 dpi Np																									
	14-54 14 dpi OPF																							T		
	14-54 35 dpi Np															A								T		
	14-54 35 dpi OPF				T																					
	14-112 1 dpi Np																									
	15-12 3 dpi Sa																									
	15-12 5 dpi Sa																									
	15-12 14 dpi OPF	T																								
	15-12 21 dpi OPF	T					T																			G
	15-12 28 dpi OPF																				T					
	15-12 35 dpi OPF		T			T												G			T	T				
	15-13 7 dpi OPF																								A	
	15-13 14 dpi OPF																							T		
	15-13 21 dpi OPF																								A	
	15-13 28 dpi OPF																								A	
	15-13 35 dpi OPF																							T		
	15-14 5 dpi Ns																									
	15-14 17 dpi OPF					T	T																			
	15-14 28 dpi OPF					T				T																
Amino acid change*				43				73	84					136				224		248					283	301
Genome region		2C																								

Genome region		3A													3B				
Nucleotide position		4285	4320	4380	4515	4572	4581	4628	4644	4645	4676	4684	4703	4718	4761	4856	4908	4911	4941
Inoculum consensus		A	T	C	T	C	C	G	C	G	A	A	C	A	A	C	T	T	C
Non-vaccinated cattle	14-20 1 dpi Np							A											
	14-25 3 dpi Np																		
	14-25 3 dpi Ves											G							
	14-25 3 dpi Se																		
	14-34 4 dpi Ns											G							
	14-34 4 dpi Sa											G							
	14-34 5 dpi Sa							A											
	14-34 6 dpi Sa											G							
	14-34 7 dpi Sa																	C	T
	14-34 8 dpi Sa																	C	T
	14-34 9 dpi Sa																	C	T
	14-34 17 dpi OPF																C		
	14-34 35 dpi OPF																C		
	14-34 35 dpi Np										G						C		
	14-44 4 dpi Ves							A											
	14-44 4 dpi Np							A											
	14-44 4 dpi Se																		
	14-47 2 dpi Sa											G							
	14-49 2 dpi Sa																		
	14-49 6 dpi Se																		
	14-49 10 dpi Np									A								C	T
	14-110 6 dpi Se																		
	14-110 6 dpi Ns																		
	14-110 8 dpi Sa																		
	14-110 14 dpi OPF							A											
	14-110 17 dpi OPF		C									G							
	14-110 21 dpi OPF											G			G				
	14-110 24 dpi OPF											G							
	14-110 28 dpi OPF											G		C					
	14-110 31 dpi OPF											G		C					
	14-110 35 dpi Np											G							
	14-110 35 dpi OPF											G							
Vaccinated cattle	14-53 3 dpi Np																		
	14-54 14 dpi OPF											G							
	14-54 35 dpi Np					T						G	T						
	14-54 35 dpi OPF											G							
	14-112 1 dpi Np																		
	15-12 3 dpi Sa											G							
	15-12 5 dpi Sa											G							
	15-12 14 dpi OPF											G							T
	15-12 21 dpi OPF								T			G							T
	15-12 28 dpi OPF				C		T					G							
	15-12 35 dpi OPF				C		T					G							
	15-13 7 dpi OPF							A											
	15-13 14 dpi OPF											G							
	15-13 21 dpi OPF	G						A											
	15-13 28 dpi OPF							A							N	N	N	N	N
	15-13 35 dpi OPF			T								G							
	15-14 5 dpi Ns											G							
	15-14 17 dpi OPF															T			
	15-14 28 dpi OPF															T			
Amino acid change*		3						117		123	133	136	142	147		40			
Genome region		3A													3B				

Genome region		3C													3D																					
Nucleotide position		5052	5061	5139	5148	5221	5295	5298	5307	5319	5442	5478	5583	5586	5596	5910	6002	6039	6063	6135	6180	6270	6279	6321	6327	6372	6402	6423	6429	6517	6558	6606	6648	6721	6819	6976
Inoculum consensus		T	C	A	C	C	C	T	C	T	A	C	C	C	A	A	C	G	T	C	C	C	C	C	T	T	T	C	C	T	T	T	C	C	T	G
Non-vaccinated cattle	14-20 1 dpi Np																																			
	14-25 3 dpi Np																																			N
	14-25 3 dpi Ves									C																										N
	14-25 3 dpi Se																																			N
	14-34 4 dpi Ns																																			
	14-34 4 dpi Sa																																			N
	14-34 5 dpi Sa																									C									C	
	14-34 6 dpi Sa																																			
	14-34 7 dpi Sa																															C				
	14-34 8 dpi Sa										G																					C				
	14-34 9 dpi Sa																															C				
	14-34 17 dpi OPF																																			
	14-34 35 dpi OPF																																			
	14-34 35 dpi Np																																			N
	14-44 4 dpi Ves																																		C	
	14-44 4 dpi Np																																		C	
	14-44 4 dpi Se																																		C	
	14-47 2 dpi Sa																																			N
	14-49 2 dpi Sa																																			
	14-49 6 dpi Se																													C						
	14-49 10 dpi Np																															C				
	14-110 6 dpi Se																																			N
	14-110 6 dpi Ns																																			
	14-110 8 dpi Sa																																			
	14-110 14 dpi OPF																																		C	
	14-110 17 dpi OPF																	A																		
	14-110 21 dpi OPF																						T	T												N
	14-110 24 dpi OPF							C															T	T												N
	14-110 28 dpi OPF							C															T	T												N
	14-110 31 dpi OPF							C												T										C					N	N
	14-110 35 dpi Np																						T													N
	14-110 35 dpi OPF							C																												
Vaccinated cattle	14-53 3 dpi Np							N	N	N						N														C						N
	14-54 14 dpi OPF																																			N
	14-54 35 dpi Np																																			N
	14-54 35 dpi OPF																																			N
	14-112 1 dpi Np																																			
	15-12 3 dpi Sa				A																															
	15-12 5 dpi Sa									C																										
	15-12 14 dpi OPF				A		T																		C											N
	15-12 21 dpi OPF				A	T	T														T				C											N
	15-12 28 dpi OPF				A										G	G	T		C																C	
	15-12 35 dpi OPF				A				T			T	T		G	G	T											T	T		C		T	T		
	15-13 7 dpi OPF																					T													C	
	15-13 14 dpi OPF																	A																		
	15-13 21 dpi OPF																					T				C									C	N
	15-13 28 dpi OPF																					T				C									C	N
	15-13 35 dpi OPF			G																							C					C		N	N	N
	15-14 5 dpi Ns									C																										
	15-14 17 dpi OPF		T											T																						A
	15-14 28 dpi OPF	C	T											T																						
Amino acid change*					66	91									3		138	150																378		463
Genome region		3C													3D																					

* Underlined amino acid changes are those residues with substitutions at two codon sites.

† Abbreviations: DPI – days post-infection, Na – nasal secretion, Np – nasopharyngeal tissue (necropsy), OPF – oropharyngeal fluid (probang cup sample), Sa – saliva, Se – serum, Ves – epithelial vesicle
